# The Echocardiographic Diagnosis of Rheumatic Heart Disease: A Review of the Performance of the World Heart Federation Criteria 2012–2023

**DOI:** 10.5334/gh.1327

**Published:** 2024-05-13

**Authors:** James Marangou, Joselyn Rwebembera, Julius Mwita, Lene Thorup, Bo Remenyi, Bruno Ramos Nascimento, Andrea Beaton, Krishna Kumar, Emmy Okello, Kate Raltson, Craig Sable, Gavin Wheaton, Nigel Wilson, Liesl Zuhlke, Cleonice Mota, Ana Mocumbi

**Affiliations:** 1 Global and Tropical Health Division, Menzies School of Health Research, Charles Darwin University, Darwin, Australia; 2 Department of Cardiology, Royal Perth Hospital, Perth, Australia; 3 Department of Cardiology, Fiona Stanley Hospital, Perth, Australia; 4 Uganda Heart Institute, Kampala, Uganda; 5 Department of Internal Medicine, University of Botswana and Princess Marina Hospital, Botswana; 6 Department of Cardiothoracic Surgery, Rigshospitalet, Copenhagen University Hospital, Denmark; 7 Department of Paediatrics, Royal Darwin Hospital, Darwin, Australia and NT Cardiac, Darwin, Australia; 8 Departamento de Clínica Médica, Faculdade de Medicina da Universidade Federal de Minas Gerais, Belo Horizonte, Brazil; 9 Serviço de Cardiologia e Cirurgia Cardiovascular, Hospital das Clínicas da Universidade Federal de Minas Gerais, Belo Horizonte, Brazil; 10 Department of Pediatrics, School of Medicine, University of Cincinnati, Cincinnati, Ohio, USA; 11 Division of Cardiology, The Heart Institute, Cincinnati Children’s Medical Center, Cincinnati, Ohio, USA; 12 Amrita Institute of Medical Sciences and Research Centre, Cochin, Kerala, India; 13 Division of Adult Cardiology, Uganda Heart Institute, Kampala, Uganda; 14 World Heart Federation, Geneva, Switzerland; 15 Division of Cardiology, Children’s National Hospital, Washington, DC, USA; 16 Women’s and Children’s Hospital, Adelaide, South Australia, Australia; 17 Green Lane Paediatric and Congenital Cardiac Services, Auckland, Te Whatu Ora, New Zealand; 18 Vice President-Extramural Research & Internal Portfolio, South Africa Medical Research Council, South Africa; 19 Division of Paediatric Cardiology, Department of Paediatrics and Child Health, University of Cape Town, Cape Town, South Africa; 20 Departamento de Pediatria, Faculdade de Medicina da Universidade Federal de Minas Gerais, Belo Horizonte, Brazil; 21 Divisão de Cardiologia Pediátrica e Fetal/Serviço de Cardiologia e Cirurgia Cardiovascular e Serviço de Pediatria, Hospital das Clínicas da Universidade Federal de Minas Gerais, Belo Horizonte, Brazil; 22 University Eduardo Mondlane, Mozambique

**Keywords:** RHD, 2012 WHF echocardiographic criteria, performance

## Abstract

**Background:**

The World Heart Federation (WHF) published the first evidence-based guidelines on the echocardiographic diagnosis of rheumatic heart disease (RHD) in 2012. These guidelines have since been applied internationally in research and clinical practice. Substantial research has assessed the utility of the 2012 WHF criteria, including its applicability in low-resource settings. This article summarises the evidence regarding the performance of the guidelines.

**Methods:**

A scoping review assessing the performance of the guidelines was performed. Cochrane, Embase, Medline, PubMed Lilacs, Sielo, and Portal BVS databases were searched for studies on the performance of the guidelines between January 2012–March 2023, and 4047 manuscripts met the search criteria, of which 34 were included. This included papers assessing the specificity, inter-rater reliability, application using hand-carried ultrasound, and modification of the criteria for simplicity. The review followed the PRISMA Extension for Scoping Reviews guideline.

**Results:**

The WHF 2012 criteria were 100% specific for definite RHD when applied in low-prevalence populations. The criteria demonstrated substantial and moderate inter-rater reliability for detecting definite and borderline RHD, respectively. The inter-rater reliability for morphological features was lower than for valvular regurgitation. When applied to hand-carried ultrasound performed by an expert, modified versions of the criteria demonstrated a sensitivity and specificity range of 79–90% and 87–93% respectively for detecting any RHD, performing best for definite RHD. The sensitivity and the specificity were reduced when performed in task-sharing but remains moderately accurate.

**Conclusion:**

The WHF 2012 criteria provide clear guidance for the echocardiographic diagnosis of RHD that is reproducible and applicable to a range of echocardiographic technology. Furthermore, the criteria are highly specific and particularly accurate for detecting definite RHD. There are limitations in applying all aspects of the criteria in specific settings, including task-sharing. This summary of evidence can inform the updated version of the WHF guidelines to ensure improved applicability in all RHD endemic regions.

## Introduction

Rheumatic heart disease (RHD) remains a leading acquired cause of cardiovascular mortality and morbidity in low- and middle-income countries and some Indigenous populations of high-income countries [1,2]. It particularly impacts a young population [3,4]. In 2006, a landmark study proved that echocardiography is ten times more sensitive than cardiac auscultation at detecting early RHD [5] (Table 1). This prompted an era of echocardiographic active case finding among asymptomatic children in RHD-endemic regions. However, different research groups utilised variable echocardiographic criteria, and thus epidemiological comparison of disease burden was difficult. In 2012 the World Heart Federation (WHF) developed and published guidelines outlining the minimum criteria for the echocardiographic diagnosis of RHD based on the best available evidence (this guideline is referred to throughout this manuscript as the WHF 2012 criteria) [6]. The WHF 2012 criteria standardised the threshold for diagnosis of RHD required for epidemiological reporting globally.

**Table 1:** Common RHD valve lesions and associated auscultation findings.


VALVE LESION	AUSCULTATION FINDINGS

Mitral regurgitation	Mid/pan-systolic murmur at apex, radiating laterally (occasionally medially/ posteriorly)

Mitral stenosis	Low-pitch, diastolic murmur at apex with patient in left lateral position.

Aortic regurgitation	Blowing decrescendo diastolic murmur at left sternal edge. Systolic murmur due to increased flow Mitral diastolic murmur (Austin Flint)

Aortic stenosis	Ejection systolic murmur over aortic region, radiating to the neck.


The criteria include functional and morphological variables of the mitral and aortic valves and diagnostic categories (definite, borderline, and normal) based on the consistency of the findings with RHD (Table 2A). Some variables are more objective than others (Table 2B and C) [6].

**Table 2:** The WHF 2012 criteria for the echocardiographic diagnosis of RHD.


**A)** 2012 WHF guidelines for diagnosis of RHD

Definite RHD (either A, B, C or D): **A.** Pathological MR and at least two morphological features of RHD of the MV **B.** MS mean gradient ≥4 mmHg **C.** Pathological AR and at least two morphological features of RHD of the AV **D.** Borderline disease of both the AV and MV Borderline RHD (either A, B, or C) **A.** At least two morphological features of RHD of the MV without pathological MR or MS **B.** Pathological MR **C.** Pathological AR

**B)** Criteria for pathological regurgitation

Pathological mitral regurgitation (All four Doppler echocardiographic criteria must be met) Seen in two views. In at least one view, jet length ≥2 cm Velocity ≥3 m/s for one complete envelope Pan-systolic jet in at least one envelope Pathological aortic regurgitation (All four Doppler echocardiographic criteria must be met) See in two views. In at least one view, jet length ≥1 cm Velocity ≥3 m/s in early diastole Pan-diastolic jet in at least one envelope

**C)** Morphological features of RHD

Features in the MV AMVL thickening ≥3 mm (age-specific) Chordal thickening Restricted leaflet motion Excessive leaflet tip motion during systole Features in the AV Irregular of focal thickening Coaptation defect Restricted leaflet motion Prolapse


WHF: World Heart Federation; RHD: Rheumatic Heart Disease; MR: Mitral Regurgitation; MV: Mitral Valve; AR: Aortic Regurgitation; AV: Aortic Valve; MS: Mitral Stenosis; AMVL: Anterior Mitral Valve Leaflet

Over the past decade of applying the criteria, RHD researchers have evaluated the performance of the criteria. The variations of the findings from these studies merited a review and synthesis of the data. We aimed to review the performance of the WHF 2012 criteria for the diagnosis of RHD in active case finding and clinical settings in RHD-endemic regions; specifically summarising the a) specificity of the criteria; b) Inter- and intra-rater reliability of the criteria; c) application of the criteria using hand-carried ultrasound; and d) modification of the criteria for simplicity and task-sharing. The results of this review have been used to inform the WHF 2023 revision of the guidelines for the echocardiographic diagnosis of RHD [7] (Table 3).

**Table 3:** Summary of the new features of the 2023 WHF guidelines [7].


Introduction of two sets of echocardiographic criteria for RHD: Screening criteria are designed principally for non-experts to apply in appropriate settings for detecting suspected cases of RHD. Confirmatory criteria are designed for experts to use to confirm a diagnosis of RHD. Classification of RHD into stages A, B, C and D based on the risk of progression to more advanced valvular heart disease; the terms ‘borderline,’ ‘definite,’ and ‘latent’ RHD are no longer recommended. Weight-based measurements for valvular regurgitation jet length. Recommendations for the management of early stages of RHD.


## Methods

This scoping review followed the methodology of the Preferred Reporting Items for Systematic Review and Meta-Analysis Extension for Scoping Reviews [8].

### Information sources and literature search

**Figure 1:**
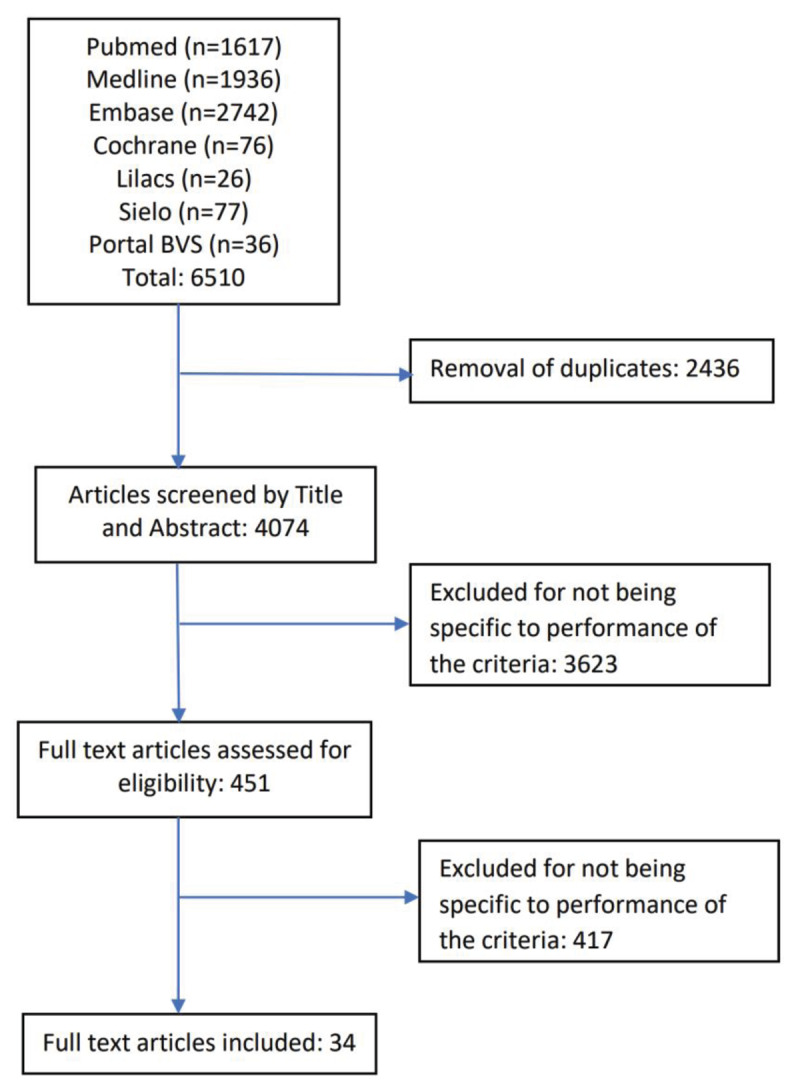
Flow diagram for literature search for studies related to performance of the 2012 WHF criteria for the echocardiographic diagnosis of RHD.

We conducted systematic electronic literature searches of Cochrane, Embase, Medline, PubMed Lilacs, Sielo, and Portal BVS databases. Languages were restricted to English, Spanish, Portuguese, and French. We used a predefined keyword search strategy (Appendix 1), to search for articles published from January 2012 to March 2023. A manual search of the reference lists of all included studies and relevant review articles supplemented the database search. We included published observational, cohort, and randomised studies utilising the 2012 WHF criteria for echocardiographic screening and diagnosis of RHD. We excluded case studies, series and studies that did not apply the 2012 WHF criteria as the reference test. All identified articles (see flow diagram, Figure 1) were collated and uploaded into EndNote X9 (Clarivate Analytics), and duplicates were removed. Two reviewers (JM and JR) independently screened titles and abstracts of all identified articles, reviewed full-text articles, and assessed their eligibility for inclusion. Disagreements between the two reviewers were resolved through a discussion and the inclusion of a third reviewer (BR).

### Data extraction

Using a predefined data extraction template, two reviewers (JR and JM) independently extracted data, including study characteristics, first author’s name, year of publication, study design, sample size, population, reference and index test details, and study outcomes.

### Data analysis and presentation

Descriptive numerical accounts were generated, including the prespecified outcomes and details of the characteristics of the studies, such as the total number of publications included, type of study design, year of publication reported, characteristics of the study populations, and study setting. Given that quantitative synthesis is inappropriate because of the considerable heterogeneity of included studies, we generated a systematic narrative synthesis of information in text and tables as appropriate.

## Results and Discussion

**Central Illustration**

- The 2012 WHF guidelines provide evidence based diagnostic criteria for RHD
- The diagnostic criteria demonstrate 100% specificity when applied in low RHD prevalence settings
- Inter-reviewer reliability of the whole criteria is at least moderate for any RHD
- Mitral regurgitation demonstrates the highest inter-reviewer reliability, whilst individual morphological features pose challenges.
- The 2012 WHF criteria can be modified for application using hand-carried ultrasound devices (without Spectral Doppler capability) and maintain diagnostic accuracy
- The diagnostic accuracy of the 2012 WHF criteria reduces when applied by non-experts using abbreviated echocardiography protocols
- A stage-based criteria can predict risk of valvular disease progression using echocardiography features.

### Performance of the 2012 criteria in low prevalence populations to determine the specificity

Echocardiography has been deemed the gold standard for the diagnosis of acute carditis and chronic rheumatic heart disease. Due to its international authorship, the WHF 2012 criteria became the default gold standard. As such, there is no comparator available to evaluate the overall accuracy of the WHF 2012 criteria. However, the specificity of the WHF 2012 criteria has been assessed through application in three screening studies of low-risk and low-prevalence populations [9,10,11]. All papers demonstrated a 0% prevalence of definite RHD when applying the WHF criteria, indicating the high specificity of the criteria for this category of RHD [9,10,11]. Clark et al. revealed a 0.8% prevalence of borderline RHD based on mitral regurgitation alone, with no morphological mitral or aortic valve features discovered. Roberts et al. found a 0.5% prevalence of borderline cases, with 0.2% demonstrating pathological mitral regurgitation, 0.2% mitral valve morphological features and 0.1% having pathological aortic regurgitation. Webb et al. also demonstrated a 0.5% prevalence of borderline RHD, based on the WHF definition of pathological mitral regurgitation [11]. Given the overlap between the findings of borderline RHD and those without RHD, some of these cases may represent false positives. This highlights the diagnostic challenge that the borderline category presents, the importance of considering individuals’ pre-test likelihood of having disease and consideration of repeat imaging over time.

### Inter-reviewer reproducibility of the 2012 WHF criteria

Studies that evaluated the inter-reviewer reliability of the 2012 WHF criteria are summarized in Table 4. Some studies assessed the reproducibility of the requirements as a whole, while others assessed the reproducibility of individual variables of the criteria.

**Table 4:** Studies of inter-reviewer reliability of the 2012 WHF criteria.


Diagnostic Feature(s)	Kane A et al. [27]	Bacquelin R et al. [13]	Culliford-Semmens et al. [14]	Remenyi et al. [15]	Beaton A et al. [16]	Scheel A et al. [12]

**Criteria as a whole**	Concordance between 3 reviewers; (6–15yo) 60%, 40%, 20%; (16–18) 100%, 90%, 90%.	k = 0.83 (95% CI 0.74–0.93) *	k = 0.57 (CI 0.44–0.7) ^2 reviewer consensus 60%3 reviewer consensus 89%	No/bor/def RHD; k = 0.49 (95% CI 0.45–0.54), def RHD; k = 0.65 (95% CI 0.59–0.7)any RHD; k = 0.6 (95% CI 0.55–0.65)	k = 1.0	Any RHD; k = 0.66 (95% CI 0.61–0.71),no/bor/def RHD; k = 0.51,def RHD: k = 0.76

**Pathological Mitral Regurgitation**	—	k = 0.92 (95% CI 0.83–1.0)	—	k = 0.60 (95% CI 0.55–0.65)	k = 1.0	k = 0.69

**Morphological Features of Mitral Valve**	—	k = 0.83 (95% CI 0.72–0.94)	—	k = 0.57 (95% CI 0.5 –0.62)	—	k = 0.57

**Mitral Regurgitation Jet Length**	—	—	—	—	ICC = 0.90	—

**AMVL Thickness**	—	k = 0.6 (95% CI 0.43–0.77)	—	k = 0.75 (95% CI 0.7–0.8)	ICC = 0.57	k = 0.6

**Restricted Motion**	—	k = 0.53 (95% CI 0.35–0.7)	—	k = 0.55 (95% CI 0.49–0.6)	k = 0.69	k = 0.7

**Chordal Thickening**	—	k = 0.62 (95% CI 0.46–0.78)	—	k = 0.54 (95% CI 0.49–0.59)	k = 0.41	k = 0.78

**Excessive Leaflet Tip Motion**	—	—	—	k = 0.63 (95% CI 0.58–0.68)	too low to assess	k = 0.74

**Pathological Aortic Regurgitation**	—	—	—	k = 0.86	—	k = 0.95

**Morphological features of Aortic Valve**	—	—	—	—	too low to assess	k = 0.89


AMVL: Anterior mitral valve leaflet, bor: Borderline RHD, def: Definite RHD k: Kappa, ICC: Intraclass correlation coefficient.

#### Whole criteria

For a firm initial diagnosis or to provide an accurate evaluation of disease progression over time, it is vital that the morphological and functional features that form the WHF 2012 criteria can be reproduced accurately and reliably between individual users. The highest quality evidence for inter-reviewer agreement for the 2012 WHF criteria is from Scheel et al. [12], who found a moderate inter-rater agreement (κ = 0.66) in the presence of any RHD. At the same time, the agreement for specific 2012 WHF classification (borderline, definite) was only fair (κ = 0.51). Across the other four studies the inter-rate reproducibility of the criteria as a whole was moderate or high, with a kappa variation of between 0.57–1.0 [13,14,15,16]. Remenyi et al. demonstrated that inter-rater reproducibility was higher for distinguishing any RHD from no RHD and definite RHD than determining borderline RHD from definite or no RHD [15].

#### Functional and morphological criteria

*Valvular regurgitation:* Scheel et al. [12] demonstrated a moderate inter-rater agreement (κ = 0.69) for pathological mitral regurgitation, which relies on the views where the longest jet length is detected. Mitral regurgitation is the most common lesion seen in screening for RHD, and it has provided the cornerstone of RHD diagnosis by echocardiography. Across other studies, mitral regurgitation has demonstrated substantial to almost perfect inter-reviewer reliability (κ = 0.6–1.0) [12,13,16]. In two studies, pathological aortic regurgitation has been assessed to have substantial or excellent inter-reviewer reliability (κ = 0.86, 0.95, respectively) [12,15].

*Morphological features:* The inter-reviewer reliability of the four morphological features of the mitral valve has been demonstrated to be moderate or substantial across several studies [12,13,15,16]. A morphologically abnormal mitral valve demonstrated an inter-reviewer reliability kappa of 0.57–0.83 across three studies [12,13,15]. Whilst some of the features are subjective, including chordal thickening, these studies demonstrated similar kappa values for inter-reviewer reliability for each feature (anterior leaflet thickening Kappa of 0.6–0.75, restricted leaflet motion kappa of 0.53–0.7, chordal thickening 0.41–0.78, excessive leaflet tip motion 0.63–0.74) [12,13,15]. The inter-rater reliability was similar for the presence of two or more morphological features (κ = 0.57) [12,15]. Overall, morphological features appear less reliable than functional features (mitral or aortic regurgitation). There is limited evidence assessing the inter-reviewer reliability of aortic valve morphological changes, partly due to the lower prevalence of aortic valve pathology compared to the mitral valve. The available evidence regarding aortic valve involvement demonstrates substantial reliability when assessing morphology overall but lacks assessment of the individual criteria [12,15].

### Intra-rater reproducibility of the 2012 WHF criteria

There has been limited evaluation of the intra-rater reliability of applying the WHF 2012 criteria. Remenyi et al. [15] reported a substantial intra-rater reliability kappa of 0.68 for differentiating no RHD from borderline and definite RHD. Like the inter-rater reliability, the intra-rater reliability was highest when distinguishing any RHD from no RHD, as opposed to separating definite RHD, borderline RHD and no RHD. This reiterates the difficulty distinguishing borderline RHD features from normal findings.

### Accuracy of the 2012 WHF criteria when applied to hand-carried echocardiography (HAND) and compared with standard echocardiography (STAND)

Hand-carried echocardiogram machines are highly portable tablet-sized devices with limited functionality, which are substantially less expensive than full-scale machines. There has been much enthusiasm regarding their role as a low-cost solution for echocardiographic active case finding in resource-constrained settings where RHD is endemic [17,18,19]. RHD active case finding with hand-carried devices has been performed with a modification of the 2012 criteria to allow for the absence of spectral Doppler function on currently available devices. We found two studies that compared the performance of HAND against STAND, both performed by experienced cardiologists or echocardiographers with a slight modification of the WHF 2012 criteria to suit the absence of the spectral Doppler function on the then-available HAND devices [20,21]. Both studies found that HAND had a moderate to high sensitivity (79%, 90.2%) and specificity (87%, 92.9%) for distinguishing between normal and RHD patients. Still, it performed best with definite RHD (sensitivity 100% and specificity 96%) [20]. The sensitivity and specificity for borderline RHD were 75% and 96%, respectively. HAND overestimated mitral valve morphologic valve abnormalities, only 66.7% specific for anterior leaflet thickness >3 mm and 79.0% for restricted leaflet motion. In both studies, the reasons for non-agreement between HAND and STAND included over-estimation of morphological features by HAND as compared to STAND and under-estimation of mitral regurgitation jet length by HAND as compared to STAND [20,21]. Four studies assessed the performance of HAND in a setting of ‘focused cardiac ultrasound,’ which comprised hand-carried echocardiography machines, non-expert echocardiographers, and variable abbreviated echocardiographic criteria [22,23,24,25]. A systematic review and meta-analysis that included the aforementioned two studies and four other studies involving non-expert echocardiographers and abbreviated echocardiographic criteria has been performed [26]. This demonstrated that HAND had the highest sensitivity and specificity for detecting definite RHD (91% and 92%, respectively) and was lower for borderline RHD (sensitivity 62%, specificity 82%) and any RHD (sensitivity 82%, specificity 90%) [26].

### Performance of simplified/abbreviated diagnostic criteria compared to the 2012 WHF criteria

#### Simplified criteria performed by experts

Multiple simplified protocols have been tested by experts using both HAND and STAND echocardiography. We identified five studies in this category of testing the performance of abbreviated echocardiographic criteria when performed by experts, summarized in Table 5. Using hand-carried echocardiography, Lu et al. [28] tested three abbreviated criteria. MR jet length ≥1.5 cm or any AI had the best combination of sensitivity and specificity for borderline or definite RHD (73.3% & 82.4%, respectively), with excellent sensitivity for definite RHD (97.9%) when compared to the gold standard of full WHF criteria by standard echocardiography. Zuhlke et al. [24] tested a highly abbreviated protocol (Hand-carried echocardiogram, one view—parasternal long-axis (PLAX), one measurement—MR jet length ≥2 cm). The sensitivity of this protocol for definite or borderline RHD was 80.8%, specificity of 100%; positive predictive value (PPV) of 100%, and negative predictive value (NPV) of 92.5%, while the performance for definite RHD was sensitivity of 92.3%, specificity of 100%, PPV 100%, NPV 98.4%. The performance dropped significantly for borderline RHD with a sensitivity of 69.2%, specificity of 100%, PPV of 100%, and NPV of 93.9%. The test reliability was 98.7% for detecting definite disease and 94.7% for detecting borderline disease. Diamantino et al. [29] also tested the abbreviated protocol of a single PLAX view and criteria of MR ⩾1.5 cm and/or any AR by hand-carried echocardiography. The sensitivity for detection of definite RHD by experts using the focussed HAND protocol was 96%. The performance was lower for any RHD and much lower for borderline RHD. Finally, Remenyi et al. [30] tested a single parasternal long-axis sweep of the heart (SPLASH) performed on standard echocardiography machines by expert cardiologists. The sensitivity, specificity, PPV, and NPV of the abbreviated protocol for detecting any RHD were 1.0, 0.95, 0.44, and 1, respectively, while the same parameters for definite RHD were 1.0, 0.94, 0.23, and 1, respectively. These studies demonstrate the potential of abbreviated criteria, a much-needed concept in population-wide active case-finding programs, particularly in RHD endemic regions.

**Table 5:** Studies of abbreviation of the WHF 2012 criteria and their accuracy when performed by an expert.


Diagnostic Feature(s)	Diamantino et al. [29]	Beaton et al. [21]	Lu et al. [28]	Remenyi et al. [30]	Zuhlke et al. [24]

Modified protocol	PLAX with colour Doppler.	11 image protocol (same as STAND)	11 image protocol, excluded CW	PLAX with sweep of the heart and color Doppler	PLAX with color Doppler

Modified criteria	Valve regurgitation only	CW replaced with regurgitation jet seen in ≥2 consecutive frames	Assessed multiple parameters that maximised sensitivity and specificity	Valve regurgitation or morphological features	

Definition of screen positive	MR ≥1.5 cm, any AR	N/A	MR ≥1.5 cm or any AR found to have the best compromise	Any MR or AR seen in 2 consecutive frames, ≥2 morph features of MV	MR ≥2 cm

Diagnosis of RHD	As per WHF	As per WHF	As per WHF	As per WHF	As per WHF

**Sensitivity** c/f ref WHF criteria by standard echocardiography for any RHD	81.10%	78.9%%	73.30%	100%	80.80%

**Specificity** c/f ref WHF criteria by standard echocardiography for any RHD	75.50%	87.2%%	82.40%	95%	100%

PPV & NPV for any RHD	63.2%; 88.5%			44%; 100%	100%, 92.5%


#### Simplified/ abbreviated criteria performed by non-experts

One of the main challenges to echocardiographic active case-finding programs in RHD endemic regions is limited expertise and human resources. As such, task sharing to non-experts with a brief training on abbreviated protocols using inexpensive hand-carried devices is becoming an increasingly popular option and subsequently has improved the implementation of such programs. The five studies of simplified WHF 2012 criteria performed by non-experts are summarized in Table 6. A simplified echocardiographic screening protocol performed by non-experts using colour Doppler to detect any mitral regurgitation or any aortic regurgitation was highly sensitive (84.2%, CI 72.1–92.5) and specific (85.6%, CI 83.9–87.1) [17]. The exact modification of the criteria has varied between studies; however, similar sensitivity and specificity has been reproduced between them (sensitivity range 70.4%–83.7%, specificity range 78.1%–90.9%). Furthermore, different cut-offs for regurgitation jet lengths impact the accuracy of the screening echocardiogram, with increasing jet lengths correlating with lower sensitivity and higher specificity. Two studies have reported that the criteria of mitral regurgitation jet length of >10 mm or any aortic regurgitation may provide the best compromise for sensitivity (82.5% [70.1–91.3], 78.9% [71.0–85.5]) and specificity (87.1% [85.5–88.6], 78.9% [77.5–80.3]) [17,31]. Most such protocols avoid morphological and spectral Doppler requirements that are difficult to train among non-expert practitioners and unavailable in hand-carried devices. Furthermore, studies have reported variation in sensitivity and specificity between non-experts receiving the same level of training, suggesting there remains an element of operator competency that impacts accuracy [25,32]. Francis and colleagues report a sensitivity and specificity of 70.4% and 78.1%, respectively, for borderline or definite RHD in a single parasternal long-axis view performed by non-experts using hand-carried devices [18]. In a subsequent study by the same group, sensitivity was improved with modified training programs for non-experts, whilst the addition of offsite expert review of the images to determine referral maintained a moderate to high specificity (sensitivity 88.4%, specificity 77.1%) [31]. Other publications have reported a similarly reasonable sensitivity (70–84%) and specificity (70–78%), which may improve with further training [19,25,33,34]. This data demonstrates the viability of abbreviated criteria in the hands of non-experts and offers promise to task-sharing in RHD active case-finding programs.

**Table 6:** Sensitivity and specificity of abbreviated 2012 WHF criteria when performed by non-experts.


	Engelman et al. [17]	Francis et al. [18]	Francis et al. [31]	Mirabel et al. [25]	Ploutz et al. [23]

Non-experts	Nurses	Nurses, Indigenous health workers, physicians	Nurses, Indigenous health workers, physicians	Nurses	Nurses

Modified protocol and criteria	12-step protocol.	Single PLAX with a sweep through the heart.	Single PLAX with a sweep through the heart.	PLAX, PSAX, A2C, A3C, A4C	PLAX, A4C, A5C

	Any MR or AR	Any MR or AR	Any MR or AR Approach 1: non-expert screen and refer. Approach 2 non-expert screen with expert off-sight review to determine referral	MR ≥2 cm or any AR	MR ≥1.5 cm and/or any AR

Gold standard	Comprehensive echo performed by Cardiologist applying 2012 WHF criteria	Comprehensive echo performed by Cardiologist applying 2012 WHF criteria	Abbreviated echo performed by expert & Comprehensive echo performed by Cardiologist applying 2012 WHF criteria	Comprehensive echo reviewed by Cardiologist applying 2012 WHF criteria	Comprehensive echo performed by Cardiologist applying 2012 WHF criteria

Findings	Sensitivity: 84.2% (95% CI 72.1–92.5)	Sensitivity: 70.4%	Approach 1: Sensitivity: 86.5%, Specificity: 61.4%	Sensitivity: 77.6%–83.7%	Sensitivity: 74.4% (95% CI 58.8–86.5)

	Specificity: 85.6% (95% CI 83.9–87.1%)	Specificity: 78.1%	Approach 2: Sensitivity: 88.4%, Specificity: 77.1%	Specificity: 90.9%–92% (variation differing between operators)	Specificity: 78.8% (95% CI 76.0–81.4)


### Score-based echocardiographic criteria for the diagnosis and predicting progression of latent RHD

In addition to studies assessing the accuracy and reproducibility of the 2012 criteria, a refined set of echocardiographic criteria has been proposed by Nunes et al. [35] that addresses one of the major criticisms of the WHF criteria—its complexity. The simplified score was based on five components of the WHF criteria, including mitral anterior valve leaflet thickening, excessive leaflet tip motion, regurgitation jet length ≥2 cm, and aortic valve focal thickening and any regurgitation (Table 7). This score developed from Brazilian and Ugandan cohorts accurately recognises definite RHD and can be utilised by non-experts using hand-carried echocardiography equipment in resource-limited environments. Additionally, this risk score performed well for predicting the adverse outcome at the time of diagnosis of latent RHD, being able to stratify risk in children with both borderline and definite RHD. Subsequently, the score was externally validated in different RHD populations with reliable prognostic accuracy [35,36,37]. Following the multi-variate analysis and to avoid multi-collinearity, two morphological features of the mitral valve were included in their final scoring criteria. These were anterior leaflet thickening (beta-coefficient 2.9) and excessive leaflet tip motion (beta-coefficient 3.1). This suggests that morphological features may be grouped into abnormalities of valve motion and abnormal valve/apparatus thickening. This important study could help form the foundation for updated criteria that will ultimately inform treatment recommendations among children with RHD detected through echocardiographic active case-finding programs based on the risk of disease progression.

**Table 7:** Simplified score as per Nunes et al. [35]) and associated points conveying risk of disease progression (low risk: 1–6, intermediate risk: 6–10, high risk >10).


VARIABLE	POINTS

**Mitral valve**	

Anterior leaflet thickening	3

Excessive leaflet tip motion	3

Regurgitation jet length ≥2 cm	6

**Aortic valve**	

Irregular or focal thickening	4

Any regurgitation	5


## Limitations

The scoping review presents all available evidence gathered within the search framework applied. Although this was thorough, it may be that a few relevant papers were not included. Quantitative analysis of the evidence was limited due to the heterogeneous nature of the papers, and as such, a narrative approach to the results has been necessary.

## Conclusion

This scoping review highlights that the WHF 2012 criteria provided the necessary resource for the widespread application of echocardiography to diagnose RHD in endemic regions globally. Subsequent research has demonstrated that the criteria are highly specific, with either moderate or substantial inter-reviewer reliability for detecting definite RHD. The evidence highlights the heterogeneity of the borderline category, including reduced reproducibility and specificity. Although the 2012 criteria include several variables that may be considered complex, they can be applied using either full-capacity or hand-carried ultrasound equipment, with some modification. Score-based approaches may reduce the complexity of the criteria. Future refinement of the requirements should focus on ensuring applicability and simplicity relevant to task-sharing and resource-constrained settings. Much of this data has been used to inform the updated WHF 2023 guidelines for the echocardiographic diagnosis of RHD, including the diagnostic criteria [7]. These guidelines also include commentary on task-sharing programs, the role of artificial intelligence and future aspects of echocardiographic screening for RHD.

## Additional File

The additional file for this article can be found as follows:

10.5334/gh.1327.s1 Appendices. Appendix 1 and 2.
