# Supplementary material for: The Echocardiographic Diagnosis of Rheumatic Heart Disease: A Review of the Performance of the World Heart Federation Criteria 2012–2023
Source: Glob Heart. 2024 May 13;19(1):47. doi: 10.5334/gh.1327 (PMC11100538; doi:10.5334/gh.1327)
Supplement: Appendices. — Appendix 1 and 2. [file gh-19-1-1327-s1.pdf]

Appendix 1: Predefined search terms

- (rheumatic OR RHD OR (rheumatic heart disease)) AND (echocardiography OR echo)
- rheumatic AND (echocardiography OR echo) AND (screening OR screen)
- (rheumatic OR RHD OR (rheumatic heart disease)) AND (echocardiography OR echo) AND mitral
- (rheumatic OR RHD OR (rheumatic heart disease)) AND (echocardiography OR echo) AND aortic
- rheumatic AND (echocardiography OR echo) AND (hand-held OR portable)
- rheumatic AND (echocardiography OR echo) AND task-shifting
- rheumatic AND (echocardiography OR echo) AND non-expert
- (echocardiography OR echo) AND (task-shifting OR non-expert)
- (echocardiography OR echo) AND ((low and middle income country) OR (resource poor))
- (rheumatic OR RHD OR (rheumatic heart disease)) AND (echocardiography OR echo) AND criteria
- (rheumatic OR RHD OR (rheumatic heart disease)) AND (echocardiography OR echo) AND guideline
- (rheumatic OR RHD OR (rheumatic heart disease)) AND (echocardiography OR echo) AND diagnos*
- (rheumatic OR RHD OR (rheumatic heart disease)) AND (echocardiography OR echo) AND pregnan*
- (rheumatic OR RHD OR (rheumatic heart disease)) AND (echocardiography OR echo) AND (child OR children OR paediatric)

Appendix 2: Echocardiographic images of RHD on hand-held ultrasound and traditional echocardiography machine.

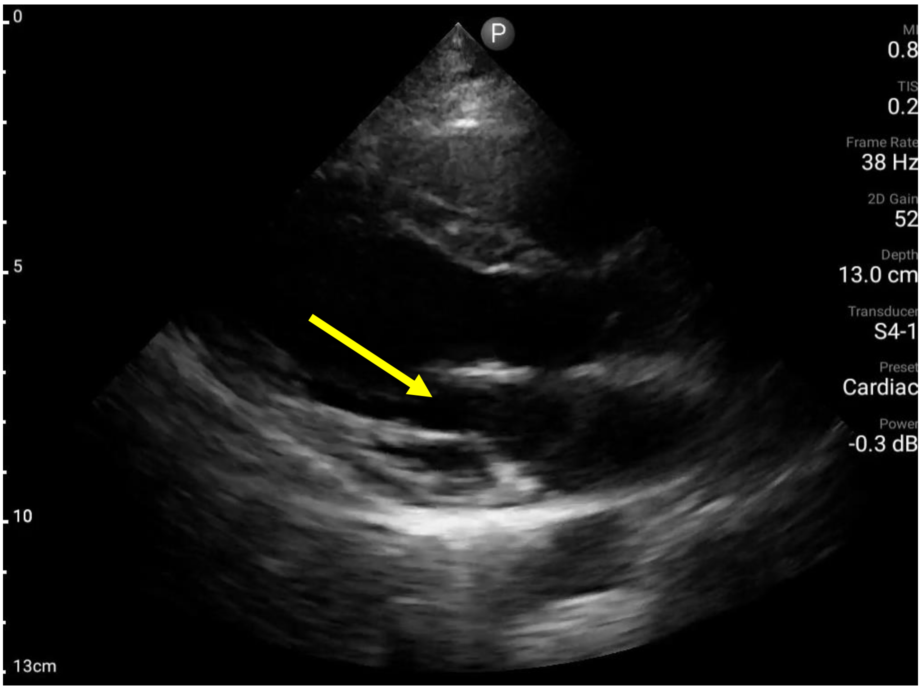

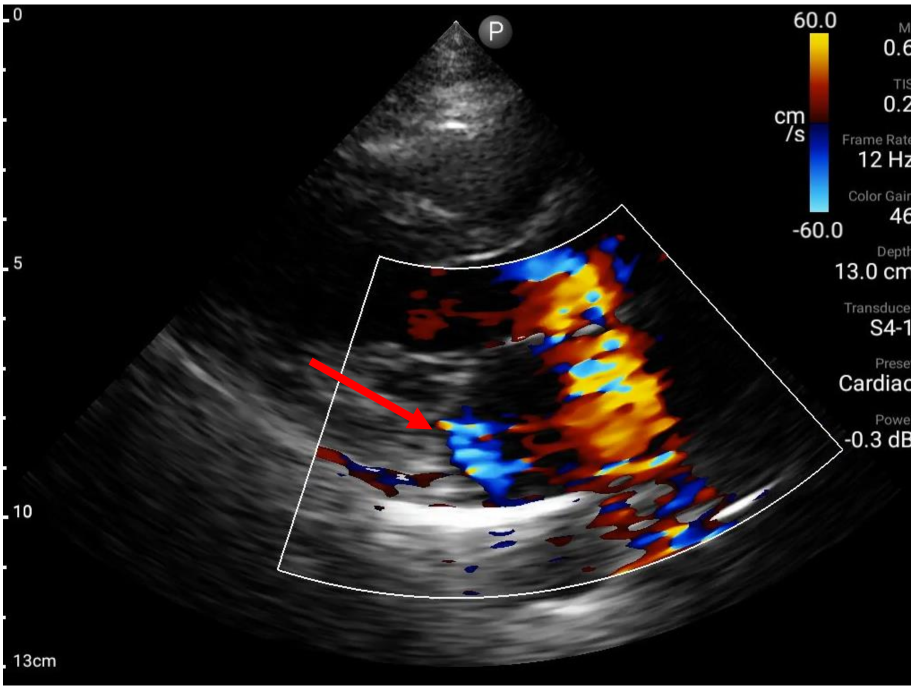

Image 1A and 1B: Handheld ultrasound image of a parasternal long axis view. Image 1A demonstrates 2D appearance of the rheumatic mitral valve with mild valve apparatus thickening (yellow arrow). Image 1B demonstrates mild mitral regurgitation (red arrow).

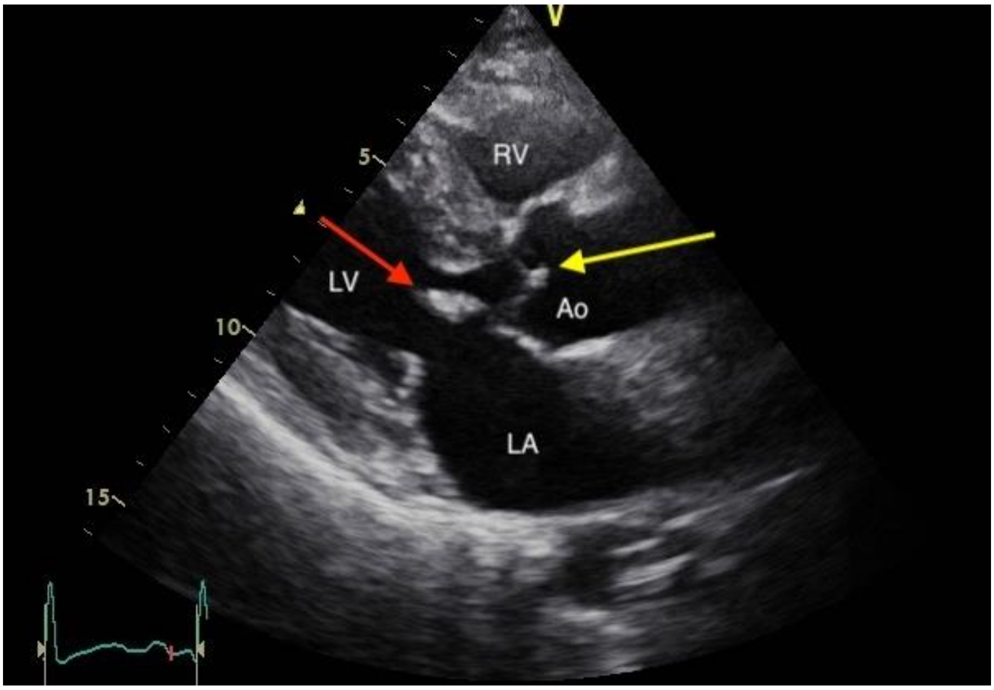

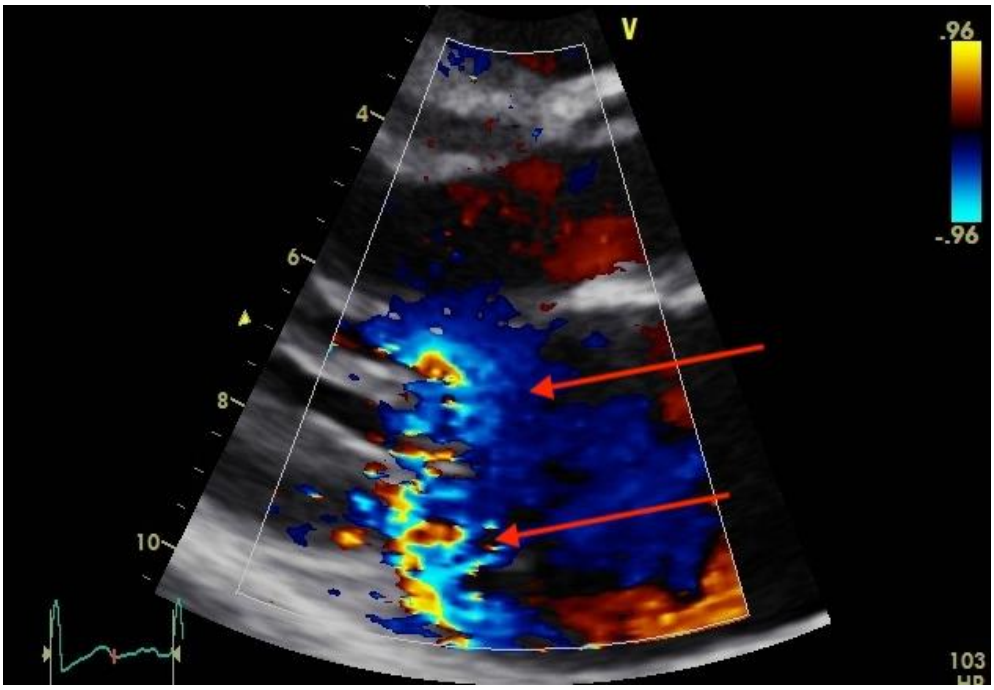

Images 2A and 2B: Traditional echocardiography image of a parasternal long axis view. Image 2A demonstrates 2D appearance of the rheumatic mitral and aortic valve with significant leaflet thickening (red and yellow arrows) and restriction of the anterior mitral valve leaflet (red arrow). Image 2B demonstrates severe eccentric mitral regurgitation (red arrows).
